# Pulmonary valve endocarditis caused by right ventricular outflow obstruction in association with sinus of valsalva aneurysm: a case report

**DOI:** 10.1186/1749-8090-3-46

**Published:** 2008-07-16

**Authors:** Katsufumi Nishida, Osamu Fukuyama, Dean S Nakamura

**Affiliations:** 1 Department of Medicine, John A. Burns School of Medicine, University of Hawaii, Honolulu, HI, USA; 2 Department of Surgery, John A. Burns School of Medicine, University of Hawaii, Honolulu, HI, USA

## Abstract

**Background:**

Right-sided infective endocarditis is uncommon. This is primarily seen in patients with intravenous drug use, pacemaker or central venous lines, or congenital heart disease. The vast majority of cases involve the tricuspid valve. Isolated pulmonary valve endocarditis is extremely rare. We report the first case of a pulmonary valve nonbacterial thrombotic endocarditis caused by right ventricular outlflow tract (RVOT) obstruction in association with a large sinus of Valsalva aneurysm.

**Case presentation:**

A 60-year-old man with a six-week history of fever, initially treated as pneumonia and sinusitis with levofloxacin, was admitted to the hospital with a new onset of a heart murmur. An echocardiogram showed thickening of the pulmonary valve suggestive of valve vegetation. A dilated aortic root and sinus of Valsalva aneurysm measuring at least 6.4 cm were also identified. The patient was empirically treated for infective endocarditis with vancomycin and gentamycin for 28 days. Four months later, the patient underwent resection of a large aortic root aneurysm and exploration of the pulmonary valve. During the surgery, vegetation of the pulmonary valve was confirmed. Microscopic pathological examination revealed fibrinous debris with acute inflammation and organizing fibrosis with chronic inflammation, compatible with a vegetation. Special stains were negative for bacteria and fungi.

**Conclusion:**

This is the first case report of a pulmonary valve nonbacterial endocarditis caused by RVOT obstruction in association with a sinus of Valsalva aneurysm. We speculate that jets created by the RVOT obstruction and large sinus of Valsalva aneurysm hitting against endothelium of the pulmonary valve is the etiology of this rare nonbacterial thrombotic endocarditis.

## Background

In the United States and Western Europe, the incidence of community-acquired native-valve endocarditis in recent studies is 1.7 to 6.2 cases per 100,000 person-years [1,2]. Right-sided endocarditis is uncommon, comprising only 5–10% of all cases of infective endocarditis [3]. This is primarily seen in patients with intravenous drug use, pacemaker or central venous lines, or congenital heart disease [4]. The majority of cases involve the tricuspid valve. Isolated pulmonary valve endocarditis is rare, with fewer than 90 cases of pulmonary valve endocarditis being previously reported [5]. The literature from 1960 to 2005 identified only 45 reported cases of pulmonary valve endocarditis in structurally normal hearts [6].

An aneurysm of the sinus of Valsalva is usually asymptomatic unless rupture occurs. However, there have been a few reported cases of unruptured sinus of Valsalva aneurysms that have presented with conduction disturbances, myocardial ischemia, and symptomatic cardiac dysfunction [7]. One of the signs of cardiac dysfunction due to sinus of Valsalva aneurysm is right ventricular outflow tract (RVOT) obstruction [8]. When it becomes large enough to cause significant outflow obstruction, symptoms such as exertional dyspnea, palpitations, and angina-like chest pain are typically observed [9]. However, cardiac structural abnormalities acquired secondary to a sinus of Valsalva aneurysm have never been reported to cause pulmonary valve endocarditis. We present the case of a 60-year-old man with nonbacterial thrombotic pulmonary valve endocarditis in association with a sinus of Valsalva aneurysm and RVOT obstruction.

## Case presentation

**Figure 1:**
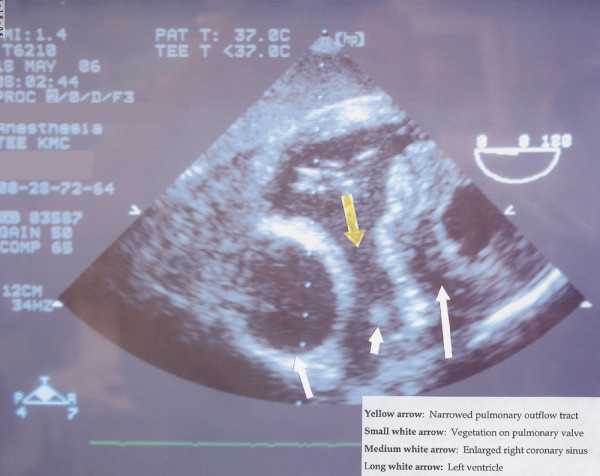
A transesophageal echocardiogram depicting an enlarged right coronary sinus (medium white arrow) and identification of the vegetation on the pulmonary valve (small white arrow).

A 60-year-old man with hypertension and type II diabetes mellitus was admitted to the local community hospital for a six-week history of low grade fever and recent development of chills and shortness of breath on October, 2005. Prior to this admission, the patient had been treated as an outpatient by his primary care physician for possible sinusitis and pneumonia with three different kinds of oral antibiotics including levofloxacin. On admission, the patient had a fever of 37 degrees centigrade and elevated white cell count of 17 × 10^9^/L. Physical examination was significant for a hyperdynamic precordium, 2+ parasternal lift, grade 2–3 out of 6 continuous murmur best heard at the left sternal boarder, and gallop at the apex. The chest x-ray showed right pleural effusion and features consistent with congestive heart failure. An echocardiogram showed a markedly dilated sinus of Valsalva, measuring at least 6.4 cm, with turbulent blood flow in a RVOT. This was felt to possibly represent a left-to-right shunt and the possibility of a ruptured sinus of Valsalva was raised. The echocardiogram also showed a vegetation on one of the pulmonary valve leaflets (figure 1). The patient was subsequently treated empirically for infective endocarditis with vancomycin and gentamycin for 28 days. During cardiac catheterization, oximetry studies showed there was no step-up in saturation in the pulmonary valve. This made the presence of a significant left-to-right shunt less likely. Aortic injection revealed significant dilation of all of the coronary sinuses. There was no jet noted from the coronary sinus to the right side of the chamber. Minimal aortic regurgitation was present. Pulmonary artery injection showed mild pulmonary insufficiency. Blood cultures did not grow any organisms.

**Figure 2:**
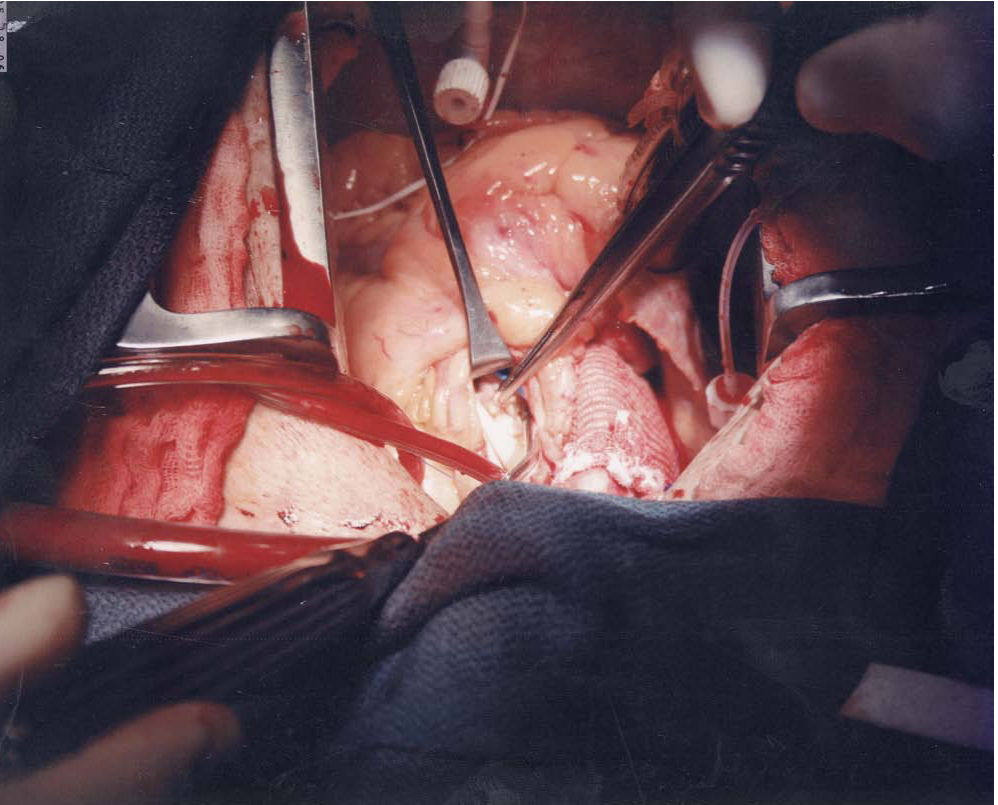
Operative photograph showing a large vegetation attached to the pulmonary valve leaflet.

**Figure 3:**
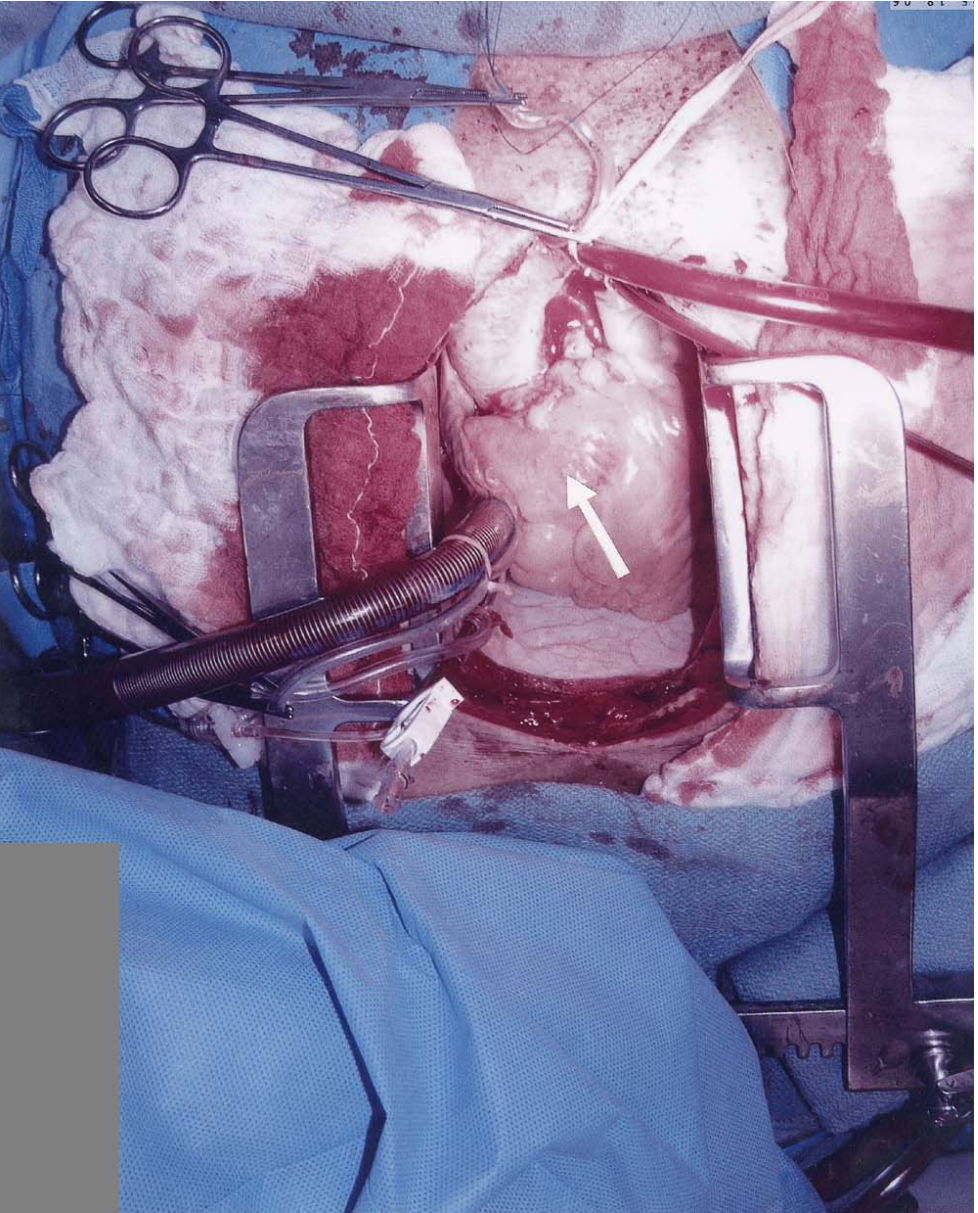
Operative photograph showing the large sinus of Valsalva aneurysm extending into the right ventricular outflow tract.

On May 2006, the patient underwent resection of a large aortic root aneurysm, replacement of the aortic valve using a 23 mm St. Jude medical valve conduit, re-implantation of left coronary artery, coronary artery bypass grafting using a saphenous vein graft to the right coronary artery, and exploration of the pulmonary valve with debridement of pulmonary valve leaflet vegetation. No evidence of a ventricular septal defect was noted during the surgery (figure 2 and 3).

The pulmonary valve vegetation was sent for pathological examination. Gross appearance consisted of multiple tan-white soft tissue fragments, measuring 1.0 × 0.8 × 0.3 cm in aggregate, the largest piece measuring 0.4 × 0.4 × 0.3 cm. Microscopic examination revealed fibrinous debris with acute inflammation and organizing fibrosis with chronic inflammation, compatible with a vegetation. Special stains were negative for bacteria and fungi.

## Discussion

Pulmonary valve infective endocarditis is uncommon. It comprises less than 2% of hospital admissions for endocarditis [10]. The low incidence of pulmonary valve infective endocarditis compared with other cardiac valves may relate to different hemodynamic pressure gradients across the valves, different frequencies of underlying congenital or acquired valvular abnormalities, lower right-sided blood oxygen content, and differences in the vascularity and endothelial lining of the valves [11]. Ramadan et al. reported that from 1960 to 1999, only 81 cases of isolated pulmonary valve endocarditis were identified. The majority of these cases (55%) occurred in patients with congenital heart disease with 36 patients (44%) having isolated pulmonary valve infective endocarditis in a normal heart [12]. In cases of right-sided endocarditis in non-drug users, 55% to 65% are caused by *S. Viridans *whereas *S. aureus *predominates in drug users [13]. Other organisms include *Neisseria, Pseudomonas, Enterococcus*, and *Haemophilus *species.

In our case, this rare nonbacterial endocarditis involving the pulmonary valve was thought to be initiated by the jet in the sub-pulmonary obstruction caused by a massively dilated aortic root that was pushing against the RVOT. We speculate that thrombus had formed on the injured endocardial surface and subsequently became infected by bacteria which had been likely irradicated by antibiotic therapy prior to pathologic examination of the vegetation. Theoretically, any acquired heart disease that causes turbulent blood flow can predispose to infective endocarditis. The case of deep sternal wires traversing the lumen of the RVOT and vegetations in the RVOT and on the pulmonary valve was reported [14]. It was also possible that a very small infundibular ventricular septal defect (VSD) could have created the jet hitting against the pulmonary valve (left-to-right shunt) resulting in the pulmonary valve endocarditis in our patient. However, the absence of a VSD as noted during surgery rules against this possibility.

## Conclusion

Isolated pulmonary valve endocarditis is difficult to recognize due to its rarity, minimal cardiac manifestations, and predominance of pulmonary infections secondary to embolization of the vegetations. This is the first case report of a pulmonary valve endocarditis caused by RVOT obstruction in association with a sinus of Valsalva aneurysm. We speculate that jets created by RVOT obstruction and large sinus of Valsalva aneurysm hitting against endothelium of the pulmonary valve is the etiology of this rare nonbacterial thrombotic endocarditis.

## Competing interests

The authors declare that they have no competing interests.

## Authors' contributions

All authors read and approved the final manuscript. KN participated in the design and drafting of the manuscript. OF cared for the patient, performed the investigation that led to the patient's diagnosis and assisted in the formulation of the manuscript. DN cared for the patient, performed the operation, prepared the images for publication and assisted in the formulation of the manuscript.

## Consent

Written consent for publication was obtained from the patient.
